# Trends in Anti‐Hyperglycemic Medicines Among US Adults With Diabetes and a History of Stroke

**DOI:** 10.1002/brb3.71921

**Published:** 2026-11-10

**Authors:** Yaojie Cai, Yu‐Jun Xiong, Tian Lv

**Affiliations:** ^1^ Department of Neurology Zhuji Affiliated Hospital of Wenzhou Medical University, Shaoxing Key Laboratory of Precision Assessment and Intelligent Prevention and Treatment of Cerebrovascular Diseases Zhuji China; ^2^ Department of Gastroenterology, Beijing Hospital, National Center For Gerontology; National Clinical Research Center For Gerontology; The Key Laboratory of Geriatrics of NHC; Institute of Geriatric Medicine Chinese Academy of Medical Sciences Beijing People's Republic of China; ^3^ Shaoxing University Medical College Shaoxing China

**Keywords:** diabetes, glucose‐lowering medications, history of stroke, medication use, MEPS

## Abstract

**Background:**

Diabetes among adults with a history of stroke is associated with increased risks of recurrent vascular events and disability and greater healthcare costs. Contemporary glucose‐lowering strategies now prioritize individualized therapy based on cardiovascular, renal, and metabolic factors over glycemic targets alone. However, national evidence on patterns of glucose‐lowering medication use for this dual‐diagnosis population is limited.

**Methods:**

We analyzed serial cross‐sectional MEPS data from 2017 to 2023 to characterize glucose‐lowering medication use among US civilian, noninstitutionalized adults aged ≥ 18 years with self‐reported diabetes and a history of stroke. Medication classes were defined via Multum codes and standardized names. Weighted prevalence with 95% CIs and linear trends were estimated. Medication use was compared between adults with and without a history of stroke using base models adjusted for sociodemographic factors and expanded models additionally adjusted for coronary disease history, annual recorded chronic kidney disease, heart failure, obesity, and approximate diabetes duration. Additional analyses used an alternative ICD‐based case definition requiring E11 and I63 records within the same survey year.

**Results:**

Among 2241 person‐years (representing 3.05 million annually), 82.4% used any glucose‐lowering agent. Metformin (52.2%), insulin (35.6%), sulfonylureas (23.1%), GLP‐1RAs (10.5%), DPP‐4is (10.5%), and SGLT2is (9.4%) were most common. GLP‐1RA/SGLT2i use rose from 8.7% to 33.8% (OR per year, 1.36; *p* < 0.001), whereas the downward trend in DPP‐4i use was not statistically significant (OR per year, 0.91; 95% CI, 0.83–1.01; *p* = 0.074). In the base models, stroke history was associated with higher odds of insulin use (OR, 1.57) and lower odds of metformin use (OR, 0.78). After additional clinical adjustment, these associations were attenuated (ORs, 1.26 and 0.86, respectively), and neither met the exploratory multiplicity‐adjusted threshold. The alternative ICD‐based cohort included 125 person‐years; trend estimates were imprecise, and some adjusted associations differed from those in the primary analysis.

**Conclusions:**

Adoption of newer agents increased markedly, yet most stroke survivors with diabetes still did not receive GLP‐1RAs or SGLT2is by 2023. The metformin and insulin associations were attenuated after additional clinical adjustment, and neither met the exploratory multiplicity‐adjusted significance threshold. Medication‐use patterns among adults with diabetes and a history of stroke warrant further investigation using data that capture clinical characteristics and treatment timing.

## Graphical abstract

In MEPS 2017'2023, 2,241 person‐years represented approximately 3.05 million U.S. adults with diabetes and a history of stroke annually. Survey‐weighted use of GLP‐1RAs or SGLT2 inhibitors increased from 8.7% to 33.8% (OR per year, 1.36; P < 0.001). Stroke‐history associations with insulin and metformin use attenuated after clinical adjustment and did not meet the exploratory multiplicity‐adjusted threshold. Despite increased uptake, approximately two‐thirds had no record of either newer drug class in 2023. Findings describe medication‐use patterns rather than causal effects.

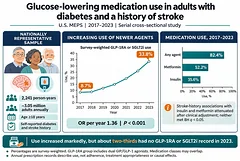

## 1 Introduction

Diabetes substantially elevates the risk of incident stroke, and when the two conditions coexist, glycemic management takes on added importance—not only for routine metabolic control but also for secondary prevention of recurrent cerebrovascular events (Maida et al. 2022). Indeed, most stroke events occurring in persons with diabetes arise against a background of longstanding metabolic derangement, and the presence of stroke, in turn, complicates diabetes care through sequelae such as physical disability, cognitive impairment, dysphagia, and depression (Yang et al. 2021). This bidirectional interplay places medication selection for glucose‐lowering therapy at the center of comprehensive secondary prevention strategies in this high‐risk subgroup.

Epidemiologically, the excess stroke hazard associated with diabetes has been consistently documented. Population‐based estimates indicate a 1.5‐ to 2‐fold elevation in stroke risk among diabetic individuals relative to their nondiabetic counterparts, with variations across studies attributable to differences in geographic setting, stroke subtype classification, diabetes duration, and case ascertainment methods (Mosenzon et al. 2023). A systematic review reported relative risk estimates spanning 1.0–2.84 for total stroke and up to 3.7 for ischemic stroke specifically (Kvitkina et al. 2023). Moreover, among patients with ischemic stroke or transient ischemic attack, diabetes is associated with increased risks of recurrent stroke, subsequent cardiovascular events, and all‐cause mortality, underscoring the importance of sustained cardiometabolic risk management in this population (Kaynak et al. 2024).

Clinical recommendations for glucose‐lowering treatment increasingly incorporate cardiovascular and kidney outcomes alongside glycemic control. Contemporary secondary prevention guidelines advocate individualized glycemic goals integrated with comprehensive risk factor modification—including blood pressure control, lipid management, smoking cessation, physical activity promotion, and antithrombotic therapy (Kleindorfer et al. 2021). For adults with type 2 diabetes and established atherosclerotic cardiovascular disease, the 2023 American Diabetes Association Standards of Care recommend an sodium–glucose cotransporter 2 inhibitor (SGLT2) inhibitor and/or a glucagon‐like peptide‐1 (GLP‐1) receptor agonist with demonstrated cardiovascular benefit as part of comprehensive cardiovascular risk reduction, independent of HbA1c (ElSayed et al. 2023). However, the two drug classes should not be regarded as interchangeable with respect to stroke prevention. A meta‐analysis of randomized trials found that GLP‐1RAs reduced stroke risk in adults with type 2 diabetes (Sattar et al. 2021). In contrast, a meta‐analysis of SGLT2 inhibitor trials did not demonstrate a significant reduction in total stroke (Pasqualotto et al. 2024). In IRIS, pioglitazone reduced the risk of stroke or myocardial infarction among insulin‐resistant patients without diabetes who had a recent ischemic stroke or transient ischemic attack (Kernan et al. 2016), though its clinical uptake is limited by concerns over weight gain, peripheral edema, bone fracture risk, and heart failure exacerbation (Azhari and Dawson 2025). Insulin and sulfonylureas are associated with hypoglycemia risk, which is an important consideration when individualizing glucose‐lowering treatment (ElSayed et al. 2023).

Prior work has described national patterns of glucose‐lowering medication use, including expenditure trends and the increasing uptake of newer drug classes (Wang et al. 2026; Fang et al. 2021). This study focused on annual medication‐use patterns among adults with diabetes and a history of stroke. We characterized class‐specific use and temporal trends from 2017 to 2023, the number of distinct medication classes recorded within a year, and adjusted comparisons with adults with diabetes without a reported history of stroke.

## 2 Materials and Methods

### 2.1 Study Design and Data Source

This serial cross‐sectional investigation drew upon seven consecutive annual waves of the Household Component of the Medical Expenditure Panel Survey spanning 2017 through 2023. The survey captures a representative segment of the US civilian, noninstitutionalized population, encompassing detailed information regarding diagnosed medical conditions, health service utilization, expenditure patterns, coverage status, sociodemographic attributes, and dispensed prescription agents. For each calendar year, the annual consolidated person‐level records were merged with the corresponding medication‐use files via a unique participant identification number. The survey design incorporates a complex, multistage probability sampling framework, with each respondent assigned individual analytic weights alongside corresponding stratum and primary sampling unit indicators to enable appropriate variance estimation. As the source materials are fully anonymized and made accessible for secondary analysis, the present work was exempt from oversight as research involving human participants.

### 2.2 Study Population

We included adults aged 18 years or older with a positive person‐level weight who reported having received a diagnosis of diabetes and having ever been diagnosed with stroke by a physician or other health care provider. The stroke measure captured a self‐reported history of stroke but lacked information on subtype, onset timing, severity, or recurrence. Accordingly, our primary sample represented adults with diabetes and a history of stroke, without restriction to a particular diabetes type, stroke subtype, or interval since stroke. For reference, we also constructed a comparison group comprising adults with diabetes who reported no history of stroke. Reliable distinction between type 1 and type 2 diabetes was not feasible from the available records. Annual medication records could not establish whether treatment preceded or followed the stroke event. The construction of the primary self‐reported cohort and the alternative ICD‐based cohort is summarized in Figure 1. The two definitions were applied separately, and the ICD‐based cohort was not treated as a subset of the primary cohort.

We additionally applied an alternative ICD‐based case definition using the annual condition files, requiring records of both E11 and I63 for the same adult within a survey year and a positive person‐level weight. This cohort was constructed independently of the primary self‐reported definition and was not required to be a subset of the primary cohort. The codes reflected conditions recorded during the survey year rather than clinically adjudicated diagnoses or the timing of stroke onset. For the alternative adjusted comparisons, the source population comprised adults with E11 records, and medication use was compared between those with and without an annual I63 record. The absence of an I63 record did not establish the absence of a history of stroke.

### 2.3 Medication Ascertainment

We categorized prescription records using the Multum therapeutic classification system, supplemented by standardized generic and brand names. The following medication groups were defined: metformin, insulin, GLP‐1RAs (including dual GLP‐1/GIP agonists), SGLT2is, DPP‐4is, sulfonylureas, thiazolidinediones, alpha‐glucosidase inhibitors, meglitinides, and amylin‐based or other glucose‐lowering therapies. For fixed‐dose combination products, each active ingredient was assigned to its respective class, so the resulting class‐specific usage proportions are not mutually exclusive across categories. At the person‐year level, we generated annual binary indicators after aggregating all prescription fills for each participant. We counted the number of distinct glucose‐lowering medication classes recorded for each person within a survey year and categorized this measure as one, two, or three or more classes. This annual count could reflect concurrent use, sequential switching, or other changes during the year and was not interpreted as a measure of treatment intensity or concurrent combination therapy. A separate composite measure captured whether a person received either a GLP‐1RA or an SGLT2i. Annual medication use was operationally defined as at least one prescription record for the relevant medication class during the survey year. This measure does not establish medication ingestion, adherence, concurrent use of multiple classes, or the clinical appropriateness of treatment. Operational case, covariate, and medication definitions are provided in Table 1.

**TABLE 1:** Operational definitions and coding decisions.

Measure	Operational definition
Primary diabetes	Adult, positive person weight; affirmative DIABDX/DIABDX_M18 response. Diabetes type is not reliably established.
Primary stroke history	Affirmative STRKDX; negative response defines comparison group. Unknown responses excluded (11 person‐years). No timing, severity, or subtype inferred.
Alternative population	Adult, positive weight, at least one annual E11 condition record.
Alternative stroke measure	At least one annual I63 record among E11‐coded adults. Comparator has no annual I63 record; it is not necessarily stroke‐free.
Coronary disease	Any yes among CHDDX, ANGIDX, and MIDX; no only if all three are no; otherwise missing.
Recorded CKD/HF/obesity	Any annual N18/I50/E66 record. Unrecorded is not validated disease absence; E66 does not represent BMI‐defined obesity.
Approximate duration	Age minus reported age at diabetes diagnosis (DIABAGED). Negative, missing, or diagnosis‐age‐greater‐than‐current‐age values excluded per 10 years in models.
Income	MEPS POVCAT: < 100%, 100–< 125%, 125–< 200%, 200–< 400%, ≥ 400% of federal poverty level.
Medication grouping	Multum therapeutic codes and generic/brand‐name rules are applied to seven PMED files; aggregation is any qualifying record within person‐year.
Metformin/insulin	Multum 214/215 or corresponding generic/brand names.
Sulfonylurea/TZD	Multum 213/271 or corresponding generic/brand names.
GLP‐1RA group	Multum 373, or named GLP‐1 agents, and tirzepatide, include dual GIP/GLP‐1 receptor agonists.
SGLT2/DPP‐4	Multum 458/371 or corresponding generic/brand names.
Other class indicators	Alpha‐glucosidase inhibitors 216; meglitinides 282; amylin/other 309 or 372, supplemented with the supplied name rules.
Any medication	Multum 99, 213–216, 271, 282, 309, 314, 371–373, 458 or specified glucose‐lowering generic‐name patterns. Broad identification does not require successful class assignment.
Annual class count	Sum of the 10 nonexclusive class flags. Separate no‐medication and unclassified‐medication categories. Not concurrent combination therapy.
Pooled design	HC‐036 h36u23, STRA9623, PSU9623, person and panel linkage; annual weights divided by seven; design declared before domain selection.

*Note*: The supplied medication algorithm was rebuilt from raw PMED files, and all 13 audited classification/count variables matched the original adult analytic data. This verifies reproducibility of the classification; it does not establish clinical indication, ingestion, or concurrent use.

### 2.4 Covariates

Covariates included continuous age, sex, races (Hispanic, non‐Hispanic White, non‐Hispanic Black, non‐Hispanic Asian, or other), annual insurance status (any private coverage, public coverage only, or uninsured), family income relative to the federal poverty level (FPL), and survey year. Family income was modeled as a categorical variable using five MEPS categories: poor/negative income (< 100% of FPL), near poor (100% to < 125%), low income (125% to < 200%), middle income (200% to < 400%), and high income (≥ 400%).

Given their clinical relevance to glucose‐lowering medication selection, we additionally included a reported history of coronary heart disease, angina, or myocardial infarction; annual recorded diagnoses of chronic kidney disease (N18), heart failure (I50), and obesity (E66); and approximate diabetes duration, calculated as age minus reported age at diabetes diagnosis. These variables were added to the sociodemographic model to examine whether the associations differed after additional clinical adjustment. The condition indicators reflected diagnoses recorded during the survey year, and the absence of a corresponding code did not establish the absence of disease. Approximate diabetes duration provided a limited proxy for disease history rather than a comprehensive measure of diabetes severity.

### 2.5 Statistical Analysis

Unweighted frequencies and survey‐weighted proportions with 95% confidence intervals were computed. Confidence intervals for medication‐use proportions were calculated using a logit transformation and survey‐design degrees of freedom. Regression confidence intervals and *p* values were calculated using the residual degrees of freedom of the survey‐weighted models. For pooled analyses of the 2017–2023 data, we used the MEPS HC‐036 Pooled Linkage Variance Estimation File (h36u23). The common variance stratum and primary sampling unit variables, STRA9624 and PSU9624, were linked to the annual records using DUPERSID and PANEL and were used without creating year‐specific strata or primary sampling units. Annual person‐level weights were divided by seven to obtain average annual population estimates. The survey design was specified using all positive‐weight records before identifying the analytic populations through domain analysis. Variances were estimated using Taylor series linearization, with a centered adjustment for singleton primary sampling units, including singleton units within analytic domains. Estimates based on fewer than 30 unweighted users were flagged as statistically unstable and were not emphasized in interpretation. Temporal trends were assessed via survey‐weighted logistic regression models incorporating calendar year as a continuous predictor. To compare medication use between adults with and without a history of stroke, we fitted two survey‐weighted logistic regression models. The base model adjusted for age, sex, race and ethnicity, insurance status, poverty category, and categorical survey year. The expanded clinical model additionally adjusted for coronary disease history, annual recorded chronic kidney disease, heart failure, obesity, and approximate diabetes duration. Complete‐case analysis was used for each model. We also repeated the base model in the clinical‐model complete‐case sample to examine the influence of sample restriction. Exploratory Benjamini–Hochberg adjustment was applied separately across the nine medication outcomes within each model family, including the base and expanded clinical models. Nominal statistical significance was defined as two‐sided *p* < 0.05, whereas *q* < 0.05 was used as the exploratory false‐discovery‐rate threshold. All analyses were carried out in R version 4.5.2 using the survey package.

For the alternative ICD‐based cohort, we recalculated pooled survey‐weighted medication‐use estimates and repeated the nine annual trend models. We also repeated the base and expanded clinical comparison models among adults with E11 records, using the presence of an annual I63 record as the exposure. These analyses used the same pooled survey variance structure and corresponding covariate specifications as the primary analyses. Given the small number of person‐years with I63 records and sparse class‐specific counts, these analyses were considered exploratory. Model convergence, exposed user counts, and confidence intervals were examined. Annual survey‐domain degrees of freedom ranged from 0 to 2, and some annual confidence intervals could not be reliably estimated. Annual medication‐use percentages were therefore presented descriptively without confidence intervals, with unweighted denominators and user counts reported in Table 2. No additional subgroup analyses were performed.

**TABLE 2:** Annual estimates for the independent E11/I63 cohort.

Medication	Year	Denominator	Users	Weighted % (descriptive)
Any glucose‐lowering medication	2017	30	25	88.4
Any glucose‐lowering medication	2018	22	20	95.0
Any glucose‐lowering medication	2019	17	14	82.5
Any glucose‐lowering medication	2020	12	11	91.3
Any glucose‐lowering medication	2021	22	20	92.0
Any glucose‐lowering medication	2022	16	15	98.2
Any glucose‐lowering medication	2023	6	5	65.8
Metformin	2017	30	18	67.8
Metformin	2018	22	12	63.3
Metformin	2019	17	8	37.8
Metformin	2020	12	8	76.7
Metformin	2021	22	17	85.1
Metformin	2022	16	12	70.1
Metformin	2023	6	4	49.8
Insulin	2017	30	15	52.1
Insulin	2018	22	9	50.4
Insulin	2019	17	7	49.3
Insulin	2020	12	7	75.8
Insulin	2021	22	7	26.0
Insulin	2022	16	5	50.8
Insulin	2023	6	2	42.5
GLP‐1RA group^a^	2017	30	0	0.0
GLP‐1RA group^a^	2018	22	0	0.0
GLP‐1RA group^a^	2019	17	1	4.3
GLP‐1RA group^a^	2020	12	1	4.1
GLP‐1RA group^a^	2021	22	4	12.9
GLP‐1RA group^a^	2022	16	2	8.9
GLP‐1RA group^a^	2023	6	2	17.9
SGLT2 inhibitors	2017	30	2	13.1
SGLT2 inhibitors	2018	22	3	17.1
SGLT2 inhibitors	2019	17	1	6.8
SGLT2 inhibitors	2020	12	1	27.4
SGLT2 inhibitors	2021	22	1	1.2
SGLT2 inhibitors	2022	16	4	37.1
SGLT2 inhibitors	2023	6	2	39.6
DPP‐4 inhibitors	2017	30	4	7.1
DPP‐4 inhibitors	2018	22	3	10.5
DPP‐4 inhibitors	2019	17	1	4.9
DPP‐4 inhibitors	2020	12	3	13.4
DPP‐4 inhibitors	2021	22	2	6.5
DPP‐4 inhibitors	2022	16	1	8.7
DPP‐4 inhibitors	2023	6	0	0.0
Sulfonylureas	2017	30	7	25.7
Sulfonylureas	2018	22	8	37.7
Sulfonylureas	2019	17	4	21.8
Sulfonylureas	2020	12	3	16.3
Sulfonylureas	2021	22	7	37.6
Sulfonylureas	2022	16	6	51.1
Sulfonylureas	2023	6	4	61.1
Thiazolidinediones	2017	30	1	0.7
Thiazolidinediones	2018	22	0	0.0
Thiazolidinediones	2019	17	0	0.0
Thiazolidinediones	2020	12	1	1.4
Thiazolidinediones	2021	22	1	2.2
Thiazolidinediones	2022	16	0	0.0
Thiazolidinediones	2023	6	0	0.0
GLP‐1RA group^a^ or SGLT2 inhibitor	2017	30	2	13.1
GLP‐1RA group^a^ or SGLT2 inhibitor	2018	22	3	17.1
GLP‐1RA group^a^ or SGLT2 inhibitor	2019	17	2	11.1
GLP‐1RA group^a^ or SGLT2 inhibitor	2020	12	2	31.5
GLP‐1RA group^a^ or SGLT2 inhibitor	2021	22	5	14.2
GLP‐1RA group^a^ or SGLT2 inhibitor	2022	16	5	38.4
GLP‐1RA group^a^ or SGLT2 inhibitor	2023	6	3	44.3

*Note*: Denominators and user counts are unweighted person‐years; percentages are survey‐weighted. All annual medication‐user counts are fewer than 30. Annual weighted percentages are presented descriptively without confidence intervals because of sparse data and unstable interval estimation. Omission of confidence intervals does not imply precision. Observed zero counts do not establish absence of medication use in the population. Annual denominators range from 6 to 30 person‐years.

^a^ The GLP‐1RA group includes dual GIP/GLP‐1 receptor agonists. Medication classes are not mutually exclusive.

## 3 Results

### 3.1 Study Participants

The primary comparison sample included 18,746 adult person‐years with self‐reported diabetes, comprising 2241 with and 16,505 without a reported history of stroke. All had complete information on the sociodemographic covariates included in the base models. The expanded clinical models included 18,022 person‐years, comprising 2115 with and 15,907 without a reported history of stroke. A total of 724 person‐years were excluded because of missing covariate information: 720 lacked valid diabetes‐duration information and six lacked coronary disease history, with two person‐years missing both variables (Table 3). The primary stroke‐history cohort included 1175 distinct respondents and represented approximately 3.05 million adults annually. Mean age was 62.1 years overall, 69.1 years in the stroke‐history group, and 61.2 years in the group without stroke history. Table 4 presents demographic and clinical characteristics for the overall diabetes population and the groups with and without stroke history.

**TABLE 3:** Covariate missingness in the diabetes comparison sample.

Covariate	Missing person‐years
Age	0
Sex	0
Race	0
Insurance	0
Poverty	0
Year	0
Stroke	0
Coronary	6
N18	0
I50	0
E66	0
Duration10	720
Any clinical‐model covariate	724
Clinical complete cases	18022
Stroke/no‐stroke clinical complete cases	2115/15,907

*Note*: Missing counts overlap: two person‐years lack both coronary history and duration. No records were excluded for the base‐model covariates. Zero missing values for condition‐code indicators reflect coding as recorded/unrecorded, not complete clinical ascertainment.

**TABLE 4:** Characteristics of adults with diabetes, by history of stroke.

Characteristic	All diabetes	Stroke history	No stroke history
Analytic sample, (*n*)	18,746	2241	16,505
Age, mean (years)	62.1 (61.6–62.5)	69.1 (68.3–69.9)	61.2 (60.7–61.7)
Approximate diabetes duration, mean (years)	12.6 (12.3–12.9)	16.2 (15.4–17.1)	12.2 (11.9–12.5)
Age group: 18—44	1932; 11.8%	52; 2.5%	1880; 12.9%
Age group: 45—64	7460; 41.1%	694; 29.8%	6766; 42.5%
Age group: 65+	9354; 47.0%	1495; 67.7%	7859; 44.5%
Sex: Male	8879; 51.1%	1107; 50.9%	7772; 51.2%
Sex: Female	9867; 48.9%	1134; 49.1%	8733; 48.8%
Race/ethnicity: Hispanic	4109; 17.1%	336; 11.5%	3773; 17.8%
Race/ethnicity: Non‐Hispanic White	9309; 57.4%	1113; 58.7%	8196; 57.3%
Race/ethnicity: Non‐Hispanic Black	3752; 15.6%	635; 21.5%	3117; 14.9%
Race/ethnicity: Non‐Hispanic Asian	897; 6.1%	53; 3.4%	844; 6.5%
Race/ethnicity: Other non‐Hispanic/multiple races	679; 3.8%	104; 4.9%	575; 3.6%
Insurance: Any private	8745; 53.7%	757; 36.9%	7988; 55.7%
Insurance: Public only	9243; 42.7%	1446; 61.6%	7797; 40.5%
Insurance: Uninsured	758; 3.6%	38; 1.5%	720; 3.8%
Family income (% federal poverty level): < 100%	4056; 14.8%	657; 20.8%	3399; 14.0%
Family income (% federal poverty level): 100%–< 125%	1248; 5.5%	182; 7.4%	1066; 5.3%
Family income (% federal poverty level): 125%–< 200%	3069; 14.9%	414; 19.5%	2655; 14.4%
Family income (% federal poverty level): 200%–< 400%	5136; 29.1%	571; 27.3%	4565; 29.3%
Family income (% federal poverty level): ≥ 400%	5237; 35.7%	417; 25.0%	4820; 37.0%
Coronary disease history	4059; 20.8%	1021; 46.3%	3038; 17.6%
Recorded chronic kidney disease (N18)	164; 0.9%	45; 1.9%	119; 0.7%
Recorded heart failure (I50)	473; 2.3%	123; 6.0%	350; 1.9%
Recorded obesity (E66)	225; 1.3%	26; 1.2%	199; 1.3%

*Note*: Continuous variables: weighted mean (95% CI); categorical variables: unweighted n; weighted %. Coronary disease is a reported history of coronary heart disease, angina, or myocardial infarction. N18, I50, and E66 indicate a code recorded during the survey year, not validated disease absence when unrecorded. Duration is age minus reported age at diabetes diagnosis. Duration is available for 18,026 person‐years; coronary disease for 18,740. Obesity coding is sparse and is not a BMI‐based measure.

### 3.2 Overall Medication Use

Any glucose‐lowering medication was used by 82.4% (95% CI, 79.3%–85.2%) of the cohort. Metformin was the most commonly recorded medication (52.2%), followed by insulin (35.6%) and sulfonylureas (23.1%). Across the entire study period, the pooled prevalence estimates for GLP‐1RAs, DPP‐4is, and SGLT2is each fell within the 9%–11% range. A composite measure capturing use of either a GLP‐1RA or an SGLT2i yielded a prevalence of 17.8% (Table 5). As individuals could be counted in multiple therapeutic categories, these class‐specific proportions are not mutually exclusive. Within a survey year, one, two, and three or more distinct glucose‐lowering medication classes were recorded in 38.6%, 27.1%, and 16.0% of cohort person‐years, respectively (Table 6). These categories describe annual medication records and do not establish concurrent use. Estimates for alpha‐glucosidase inhibitors, meglitinides, and amylin‐based or other agents were unstable and are not reported for the primary cohort.

**TABLE 5:** Annual medication‐use prevalence in the primary cohort.

Medication/category	*n*	Weighted % (95% CI)	Precision flag
Any glucose‐lowering medication	1831	82.4 (79.3–85.2)	
Metformin	1145	52.2 (48.6–55.7)	
Insulin	822	35.6 (32.0–39.4)	
GLP‐1RA group^a^	244	10.5 (8.6–12.7)	
SGLT2 inhibitors	198	9.4 (7.7–11.5)	
DPP‐4 inhibitors	240	10.5 (8.4–13.0)	
Sulfonylureas	482	23.1 (19.9–26.5)	
Thiazolidinediones	76	3.6 (2.4–5.4)	
GLP‐1RA group^a^ or SGLT2 inhibitor	393	17.8 (15.4–20.4)	
Alpha‐glucosidase inhibitors	0	0.0 (not estimated)	Sparse
Meglitinides	0	0.0 (not estimated)	Sparse
Amylin/other classified agents	0	0.0 (not estimated)	Sparse

*Note*: All *n* values are unweighted person‐years. Percentages and confidence intervals account for the pooled complex survey design. A person‐year may contribute to multiple medication groups; percentages across drug classes need not sum to 100%.

^a^ The GLP‐1RA group includes dual GIP/GLP‐1 receptor agonists. These groups do not establish a cardiovascular indication or concurrent use.

Sparse denotes fewer than 30 observed users; such estimates are descriptive and should be interpreted cautiously. A zero count does not establish absence of use in the population.

**TABLE 6:** Number of distinct medication classes recorded within a year.

Medication/category	*n*	Weighted % (95% CI)	Precision flag
No recorded glucose‐lowering medication	410	17.6 (14.8–20.7)	
First class	869	38.6 (35.1–42.1)	
Second classes	586	27.1 (24.1–30.2)	
Third or more classes	361	16.0 (13.5–18.9)	
Medication recorded; class unclassified	15	0.8 (0.4–1.6)	Sparse

*Note*: All *n* values are unweighted person‐years. Percentages and confidence intervals account for the pooled complex survey design. A person‐year may contribute to multiple medication groups; percentages across drug classes need not sum to 100%. Sparse denotes fewer than 30 observed users; such estimates are descriptive and should be interpreted cautiously. A zero count does not establish absence of use in the population. The denominator is all 2241 primary‐cohort person‐years. The mutually exclusive categories include 15 person‐years with recorded medication use but no classified class. Annual class counts cannot distinguish simultaneous treatment from switching.

^*^The GLP‐1RA group includes dual GIP/GLP‐1 receptor agonists. These groups do not establish a cardiovascular indication or concurrent use.

### 3.3 Temporal Trends

Use of the GLP‐1 receptor agonist group or SGLT2 inhibitors increased from 8.7% in 2017 to 33.8% in 2023. The odds ratio per year was 1.36 (95% CI, 1.24–1.48; *p* < 0.001). Corresponding estimates were 1.23 (1.11–1.35) for the GLP‐1 receptor agonist group and 1.50 (1.32–1.70) for SGLT2 inhibitors; both *p* values were < 0.001. These three trends also met *q* < 0.05. The DPP‐4 inhibitor estimate was 0.91 (0.83–1.01; *p* = 0.074), and the remaining full‐period trends were not statistically significant (Table 7; Figure 2). Annual estimates and sparse‐data flags are provided in Table 8.

**TABLE 7:** Period‐specific prevalence and annual trend tests in the primary cohort.

Medication	2017–2019, (%)	2020, (%)	2021–2023, (%)	OR/year (95% CI)	*p*	BH q
Any glucose‐lowering medication	85.6	80.6	79.9	0.93 (0.86–1.01)	0.078	0.141
Metformin	53.7	56.5	49.0	0.96 (0.90–1.02)	0.189	0.262
Insulin	36.3	34.9	35.1	0.98 (0.91–1.04)	0.466	0.524
GLP‐1RA group^a^	7.2	8.6	14.4	1.23 (1.11–1.35)	< 0.001	< 0.001
SGLT2 inhibitors	3.4	7.7	16.1	1.50 (1.32–1.70)	< 0.001	< 0.001
DPP‐4 inhibitors	11.6	13.1	8.4	0.91 (0.83–1.01)	0.074	0.141
Sulfonylureas	25.9	20.2	21.3	0.95 (0.87–1.03)	0.203	0.262
Thiazolidinediones	3.6	2.8	3.8	1.01 (0.81–1.25)	0.933	0.933
GLP‐1RA group^a^ or SGLT2 inhibitor	9.8	15.0	26.8	1.36 (1.24–1.48)	< 0.001	< 0.001

*Note*: Period denominators: 986, 358, and 897 person‐years, respectively. Trends use calendar year as a continuous predictor across all seven annual observations, rather than treating these three descriptive periods as the predictor. BH q values were added during revision across nine trend outcomes. All *n* values are unweighted person‐years. Percentages and confidence intervals account for the pooled complex survey design. A person‐year may contribute to multiple medication groups; percentages across drug classes need not sum to 100%.

^a^ The GLP‐1RA group includes dual GIP/GLP‐1 receptor agonists. These groups do not establish a cardiovascular indication or concurrent use.

**FIGURE 1:**
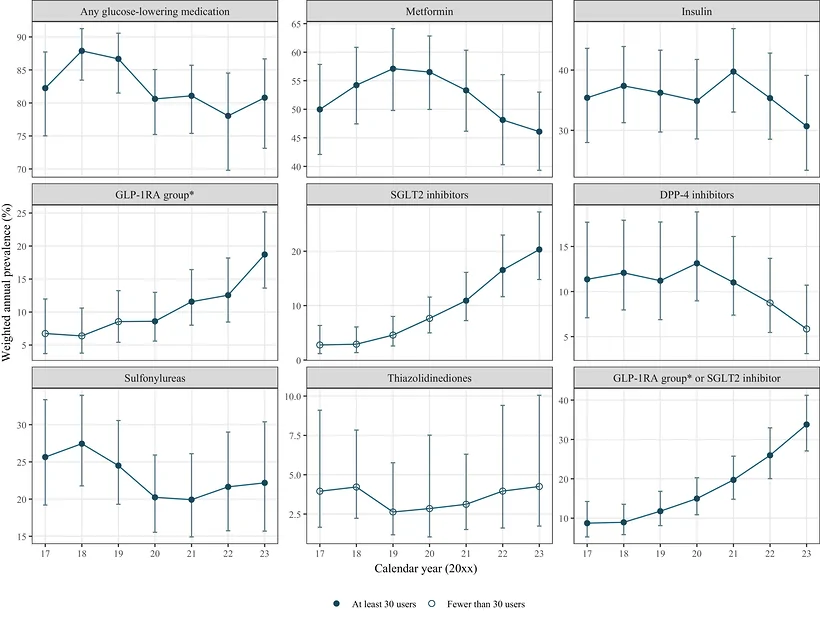
Selection of person‐year samples for the primary self‐reported and alternative ICD‐based analyses, MEPS 2017–2023. The definitions were applied independently. Counts represent unweighted person‐years, and complete‐case sample sizes are shown for the corresponding adjusted analyses. Cross‐sectional annual records were pooled; this diagram does not describe a longitudinal follow‐up cohort.

**TABLE 8:** Annual estimates underlying Figure 1.

Medication	Year	Denominator	Users	Weighted % (95% CI)	Flag
Any glucose‐lowering medication	2017	341	280	82.2 (75.0–87.7)	
Any glucose‐lowering medication	2018	336	286	87.9 (83.5–91.3)	
Any glucose‐lowering medication	2019	309	264	86.7 (81.5–90.6)	
Any glucose‐lowering medication	2020	358	286	80.6 (75.2–85.1)	
Any glucose‐lowering medication	2021	362	284	81.1 (75.4–85.7)	
Any glucose‐lowering medication	2022	288	228	78.0 (69.8–84.5)	
Any glucose‐lowering medication	2023	247	203	80.8 (73.1–86.7)	
Metformin	2017	341	168	50.0 (42.1–57.9)	
Metformin	2018	336	174	54.2 (47.4–60.8)	
Metformin	2019	309	172	57.1 (49.8–64.1)	
Metformin	2020	358	193	56.5 (50.0–62.8)	
Metformin	2021	362	184	53.3 (46.2–60.3)	
Metformin	2022	288	139	48.1 (40.3–56.1)	
Metformin	2023	247	115	46.1 (39.3–53.0)	
Insulin	2017	341	128	35.4 (28.0–43.6)	
Insulin	2018	336	127	37.4 (31.3–43.9)	
Insulin	2019	309	115	36.2 (29.7–43.3)	
Insulin	2020	358	123	34.9 (28.6–41.8)	
Insulin	2021	362	139	39.8 (33.0–46.9)	
Insulin	2022	288	108	35.3 (28.5–42.8)	
Insulin	2023	247	82	30.7 (23.4–39.1)	
GLP‐1RA group^a^	2017	341	22	6.7 (3.7–12.0)	Sparse
GLP‐1RA group^a^	2018	336	21	6.4 (3.8–10.6)	Sparse
GLP‐1RA group^a^	2019	309	29	8.5 (5.4–13.2)	Sparse
GLP‐1RA group^a^	2020	358	34	8.6 (5.6–13.0)	
GLP‐1RA group^a^	2021	362	46	11.6 (8.0–16.4)	
GLP‐1RA group^a^	2022	288	44	12.6 (8.5–18.2)	
GLP‐1RA group^a^	2023	247	48	18.7 (13.6–25.2)	
SGLT2 inhibitors	2017	341	10	2.8 (1.2–6.3)	Sparse
SGLT2 inhibitors	2018	336	11	2.9 (1.4–6.1)	Sparse
SGLT2 inhibitors	2019	309	15	4.6 (2.6–8.0)	Sparse
SGLT2 inhibitors	2020	358	29	7.7 (5.0–11.6)	Sparse
SGLT2 inhibitors	2021	362	39	10.9 (7.3–16.1)	
SGLT2 inhibitors	2022	288	44	16.5 (11.6–23.0)	
SGLT2 inhibitors	2023	247	50	20.3 (14.8–27.2)	
DPP‐4 inhibitors	2017	341	38	11.4 (7.1–17.7)	
DPP‐4 inhibitors	2018	336	43	12.1 (8.0–17.9)	
DPP‐4 inhibitors	2019	309	31	11.2 (6.9–17.7)	
DPP‐4 inhibitors	2020	358	43	13.1 (9.0–18.8)	
DPP‐4 inhibitors	2021	362	43	11.0 (7.4–16.1)	
DPP‐4 inhibitors	2022	288	28	8.7 (5.5–13.7)	Sparse
DPP‐4 inhibitors	2023	247	14	5.9 (3.1–10.7)	Sparse
Sulfonylureas	2017	341	84	25.6 (19.2–33.4)	
Sulfonylureas	2018	336	85	27.4 (21.8–34.0)	
Sulfonylureas	2019	309	73	24.5 (19.3–30.6)	
Sulfonylureas	2020	358	69	20.2 (15.5–25.9)	
Sulfonylureas	2021	362	68	19.9 (14.9–26.1)	
Sulfonylureas	2022	288	56	21.6 (15.7–29.0)	
Sulfonylureas	2023	247	47	22.2 (15.7–30.4)	
Thiazolidinediones	2017	341	11	4.0 (1.7–9.1)	Sparse
Thiazolidinediones	2018	336	14	4.2 (2.2–7.8)	Sparse
Thiazolidinediones	2019	309	9	2.6 (1.2–5.8)	Sparse
Thiazolidinediones	2020	358	11	2.8 (1.0–7.5)	Sparse
Thiazolidinediones	2021	362	12	3.1 (1.5–6.3)	Sparse
Thiazolidinediones	2022	288	8	4.0 (1.6–9.4)	Sparse
Thiazolidinediones	2023	247	11	4.3 (1.7–10.1)	Sparse
GLP‐1RA group^a^ or SGLT2 inhibitor	2017	341	30	8.7 (5.2–14.2)	
GLP‐1RA group^a^ or SGLT2 inhibitor	2018	336	31	8.9 (5.8–13.5)	
GLP‐1RA group^a^ or SGLT2 inhibitor	2019	309	40	11.8 (8.1–16.8)	
GLP‐1RA group^a^ or SGLT2 inhibitor	2020	358	57	15.0 (10.9–20.3)	
GLP‐1RA group^a^ or SGLT2 inhibitor	2021	362	72	19.7 (14.8–25.8)	
GLP‐1RA group^a^ or SGLT2 inhibitor	2022	288	80	26.0 (20.0–32.9)	
GLP‐1RA group^a^ or SGLT2 inhibitor	2023	247	83	33.8 (27.1–41.2)	

*Note*: All *n* values are unweighted person‐years. Percentages and confidence intervals account for the pooled complex survey design. A person‐year may contribute to multiple medication groups; percentages across drug classes need not sum to 100%.

^a^ The GLP‐1RA group includes dual GIP/GLP‐1 receptor agonists. These groups do not establish a cardiovascular indication or concurrent use.

### 3.4 Comparison With Adults With Diabetes Without a Reported History of Stroke

In the base model, stroke history was associated with lower odds of metformin use and higher odds of insulin use; both comparisons met the exploratory Benjamini–Hochberg threshold (*q* = 0.005 and *q* < 0.001, respectively). After additional clinical adjustment, the metformin association was attenuated and was no longer statistically significant (adjusted OR, 0.86; 95% CI, 0.74–1.01; *p* = 0.063). The insulin association was also attenuated (adjusted OR, 1.26; 95% CI, 1.05–1.53; *p* = 0.015), although it did not meet the exploratory Benjamini–Hochberg threshold (*q* = 0.131). None of the nine clinical‐model comparisons met *q* < 0.05 (Table 9; Figure 3). The base and clinical models included 18,746 and 18,022 person‐years, respectively. Results from the base model restricted to the same clinical complete‐case sample are presented in Table 10.

**TABLE 9:** Associations between stroke history and annual medication use.

Medication	Base aOR (95% CI)	*p*	Clinical aOR (95% CI)	*p*	Clinical BH q	Base BH q
Any glucose‐lowering medication	0.99 (0.80–1.23)	0.960	0.98 (0.79–1.21)	0.836	0.975	0.996
Metformin	0.78 (0.67–0.90)	0.001	0.86 (0.74–1.01)	0.063	0.284	0.005
Insulin	1.57 (1.32–1.87)	< 0.001	1.26 (1.05–1.53)	0.015	0.131	< 0.001
GLP‐1RA group^a^	0.91 (0.72–1.15)	0.410	0.82 (0.64–1.06)	0.123	0.370	0.921
SGLT2 inhibitors	1.12 (0.88–1.43)	0.358	1.02 (0.79–1.32)	0.864	0.975	0.921
DPP‐4 inhibitors	0.98 (0.76–1.26)	0.852	1.00 (0.77–1.29)	0.989	0.989	0.996
Sulfonylureas	1.02 (0.84–1.24)	0.811	1.06 (0.87–1.30)	0.554	0.975	0.996
Thiazolidinediones	1.00 (0.64–1.56)	0.996	0.96 (0.62–1.50)	0.866	0.975	0.996
GLP‐1RA group^a^ or SGLT2 inhibitor	1.03 (0.85–1.24)	0.755	0.93 (0.76–1.14)	0.503	0.975	0.996

*Note*: Base model (*n* = 18,746): continuous age, sex, race/ethnicity, insurance, five income categories, and categorical survey year. Clinical model (*n* = 18,022): base covariates plus coronary disease, annual N18/I50/E66 indicators, and approximate diabetes duration (per 10 years). Reference: diabetes without a history of stroke. Both models are descriptive‐adjusted associations. Benjamini–Hochberg adjustment was applied separately across the nine medication outcomes in the base and clinical models. *p* values are nominal; *q* values are multiplicity‐adjusted. The base‐model metformin and insulin comparisons met *q* < 0.05, whereas none of the clinical‐model comparisons met this threshold. The insulin comparison is nominally significant but not significant after BH adjustment. All *n* values are unweighted person‐years. Percentages and confidence intervals account for the pooled complex survey design. A person‐year may contribute to multiple medication groups; percentages across drug classes need not sum to 100%.

^a^ The GLP‐1RA group includes dual GIP/GLP‐1 receptor agonists. These groups do not establish a cardiovascular indication or concurrent use.

**FIGURE 2:**
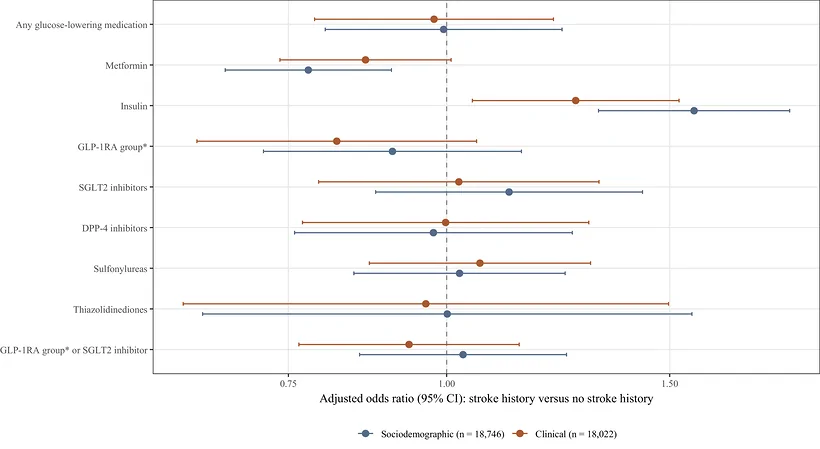
Survey‐weighted annual prevalence of selected glucose‐lowering medication classes among US adults with diabetes and a history of stroke, MEPS 2017–2023. Bars indicate 95% confidence intervals. Open symbols indicate estimates based on fewer than 30 unweighted medication users. *GLP‐1RA group includes dual GIP/GLP‐1 receptor agonists. Classes may overlap within a year. Annual estimates use the full primary cohort; a zero count has no plotted confidence interval.

**TABLE 10:** Base and clinical models fitted to the same complete‐case sample.

Medication	Base on same *n*: aOR (95% CI)	Clinical: aOR (95% CI)
Any glucose‐lowering medication	1.03 (0.83–1.27)	0.98 (0.79–1.21)
Metformin	0.77 (0.66–0.90)	0.86 (0.74–1.01)
Insulin	1.58 (1.33–1.87)	1.26 (1.05–1.53)
GLP‐1RA group	0.86 (0.68–1.10)	0.82 (0.64–1.06)
SGLT2 inhibitors	1.11 (0.86–1.42)	1.02 (0.79–1.32)
DPP‐4 inhibitors	1.03 (0.80–1.32)	1.00 (0.77–1.29)
Sulfonylureas	1.05 (0.87–1.28)	1.06 (0.87–1.30)
Thiazolidinediones	0.89 (0.58–1.36)	0.96 (0.62–1.50)
GLP‐1RA group or SGLT2 inhibitor	1.00 (0.82–1.21)	0.93 (0.76–1.14)

*Note*: Both models use 18,022 person‐years. This comparison separates changes associated with covariate adjustment from changes associated with sample restriction; it does not establish a causal explanation.

### 3.5 Analyses Using an Alternative ICD‐Based Case Definition

The alternative ICD‐based cohort, defined by E11 and I63 records within the same survey year, included 125 person‐years, corresponding to an average annual population of approximately 161,000 US adults. Of these, 63 person‐years also met the primary self‐reported definition, whereas 62 met only the alternative definition. Conversely, 2178 person‐years met only the primary definition (Figures 1 and 4). Pooled medication‐use estimates are presented in Table 11. None of the nine repeated annual trend tests reached nominal statistical significance. The OR per year was 1.78 (95% CI, 0.99–3.18; *p* = 0.052) for GLP‐1RA use and 1.32 (95% CI, 0.82–2.11; *p* = 0.232) for the GLP‐1RA/SGLT2i composite measure. The trend estimates were imprecise (Table 12). Annual sample sizes ranged from 6 to 30 person‐years, and all annual medication‐user counts were fewer than 30. Annual weighted percentages are presented descriptively without confidence intervals because interval estimation was unstable in the sparse annual survey domains (Table 2; Figure 5). These percentages should not be interpreted as precise national estimates, and apparent year‐to‐year fluctuations should not be taken as evidence of temporal trends.

**TABLE 11:** Descriptive estimates under the independent E11/I63 definition.

Medication/category	*n*	Weighted % (95% CI)	Precision flag
Any glucose‐lowering medication	110	89.3 (77.0–95.4)	
Metformin	79	64.8 (49.5–77.6)	
Insulin	52	49.4 (34.5–64.5)	
GLP‐1RA group^a^	10	5.7 (2.2–14.0)	Sparse
SGLT2 inhibitors	14	19.3 (9.4–35.4)	Sparse
DPP‐4 inhibitors	14	7.6 (3.5–15.8)	Sparse
Sulfonylureas	39	35.2 (22.1–51.1)	
Thiazolidinediones	3	0.6 (0.1–2.8)	Sparse
GLP‐1RA group^a^ or SGLT2 inhibitor	22	22.5 (12.1–38.0)	Sparse

*Note*: All *n* values are unweighted person‐years. Percentages and confidence intervals account for the pooled complex survey design. A person‐year may contribute to multiple medication groups; percentages across drug classes need not sum to 100%. Sparse denotes fewer than 30 observed users; such estimates are descriptive and should be interpreted cautiously. A zero count does not establish absence of use in the population. *N* = 125 person‐years; 63 overlap the primary cohort, 62 meet only the alternative definition, and 2178 meet only the primary definition. Repeated annual trends and adjusted comparisons are reported in Tables 11, 12, 13. Sparse estimates do not validate the primary findings.

**TABLE 12:** Repeated annual trend models in the independent E11/I63 cohort.

Medication	Users/125	OR/year (95% CI)	*p*	BH q	Sparse^a^
Any glucose‐lowering medication	110	0.93 (0.53–1.62)	0.788	0.970	Yes
Metformin	79	1.03 (0.71–1.49)	0.862	0.970	No
Insulin	52	0.94 (0.70–1.26)	0.671	0.970	No
GLP‐1RA group^a^	10	1.78 (0.99–3.18)	0.052	0.469	Yes
SGLT2 inhibitors	14	1.28 (0.76–2.14)	0.327	0.736	Yes
DPP‐4 inhibitors	14	0.93 (0.64–1.37)	0.714	0.970	Yes
Sulfonylureas	39	1.23 (0.84–1.81)	0.263	0.736	No
Thiazolidinediones	3	1.01 (0.51–1.98)	0.983	0.983	Yes
GLP‐1RA group^a^ or SGLT2 inhibitor	22	1.32 (0.82–2.11)	0.232	0.736	Yes

*Note*: All nine models converged.

^a^ Sparse means fewer than 30 exposed users or fewer than 30 exposed nonusers. Estimates are exploratory and may be unstable despite convergence. GLP‐1RA group includes dual GIP/GLP‐1 receptor agonists. No trend meets nominal *p* < 0.05. These are alternative‐definition analyses, not a stricter subset analysis.

**FIGURE 3:**
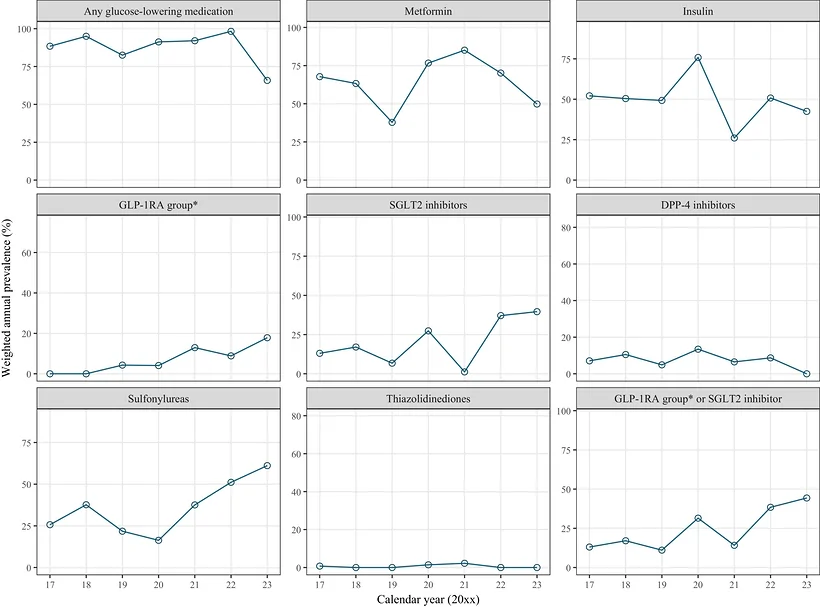
Adjusted associations between stroke history and use of glucose‐lowering medication classes among US adults with diabetes, MEPS 2017–2023. Points are adjusted odds ratios, and horizontal lines are 95% confidence intervals; the dashed vertical line denotes an odds ratio of 1.0. The reference group is adults with diabetes and no history of stroke. The base models adjusted for age, sex, race and ethnicity, insurance status, poverty category, and survey year. The expanded clinical models additionally adjusted for coronary disease history, annual recorded chronic kidney disease, heart failure, obesity, and approximate diabetes duration. Models account for the pooled complex survey design. Clinical adjustment adds coronary disease, recorded CKD, heart failure and obesity, and approximate diabetes duration. *Includes dual GIP/GLP‐1 receptor agonists. No clinical‐model comparison met BH *q* < 0.05. These estimates describe associations and do not identify effects of stroke history.

The alternative comparison population comprised 17,012 person‐years with E11 records, including 125 with and 16,887 without I63 records. The expanded clinical model included 15,763 complete cases, of which 115 had I63 records. Some adjusted associations differed from the primary analysis, including higher odds of insulin use (adjusted OR, 2.48; 95% CI, 1.45–4.24) and SGLT2i use (adjusted OR, 2.92; 95% CI, 1.36–6.28). The lower thiazolidinedione‐use estimate was based on only three exposed users and warrants particular caution (Table 13; Figure 6). These comparisons concern annual recorded diagnoses and are not equivalent to comparisons based on self‐reported stroke history.

**TABLE 13:** Repeated adjusted comparisons under the annual ICD definition.

Medication	Exposed users, clinical	Base aOR (95% CI)	Clinical aOR (95% CI)	Clinical *p*	Clinical BH q
Any glucose‐lowering medication	101	0.84 (0.41–1.70)	0.75 (0.36–1.57)	0.446	0.502
Metformin	72	1.05 (0.63–1.76)	1.08 (0.60–1.97)	0.789	0.789
Insulin	50	2.28 (1.34–3.86)	2.48 (1.45–4.24)	< 0.001	0.008
GLP‐1RA group^*^	9	0.45 (0.20–1.04)	0.47 (0.21–1.04)	0.063	0.141
SGLT2 inhibitors	14	2.63 (1.23–5.62)	2.92 (1.36–6.28)	0.006	0.019
DPP‐4 inhibitors	14	0.61 (0.30–1.23)	0.66 (0.32–1.34)	0.250	0.321
Sulfonylureas	34	1.66 (0.94–2.92)	1.53 (0.83–2.83)	0.174	0.313
Thiazolidinediones	3	0.14 (0.04–0.49)	0.16 (0.04–0.54)	0.003	0.016
GLP‐1RA group^*^ or SGLT2 inhibitor	21	1.38 (0.71–2.67)	1.51 (0.75–3.03)	0.249	0.321

*Note*: Source population: 17,012 adult person‐years with E11 records (125 with I63, 16,887 without I63). Base complete cases: 17,006 (six exclusions). Clinical complete cases: 15,763 (1249 exclusions), including 115 with I63 and 15,648 without I63. Model covariates match Table 5; exposure is annual I63 presence, not self‐reported stroke history. All 18 comparison models converged. Thiazolidinedione estimates are based on only three exposed users and should not be interpreted as a stable association. The absence of a recorded I63 code does not demonstrate an absence of stroke history.

^a^ The GLP‐1RA group includes dual GIP/GLP‐1 receptor agonists. These groups do not establish a cardiovascular indication or concurrent use.

**FIGURE 4:**
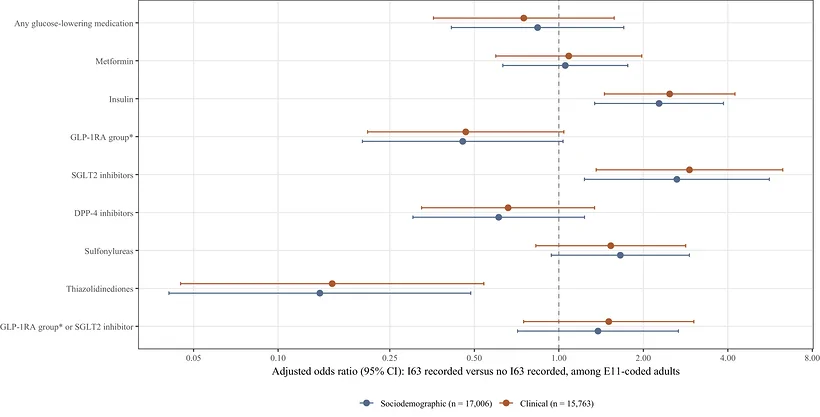
Overlap between the primary self‐reported and alternative ICD‐based cohorts, MEPS 2017–2023. The primary cohort included 2241 person‐years, and the alternative cohort included 125 person‐years. The definitions overlapped in 63 person‐years; 2178 met only the primary definition and 62 met only the alternative definition. Counts are person‐years. Circle areas are schematic, not proportional to sample size.

**FIGURE 5:**
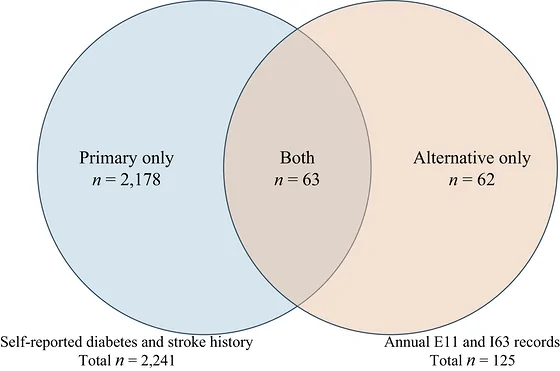
Survey‐weighted annual prevalence of selected glucose‐lowering medication classes in the alternative ICD‐based cohort defined by E11 and I63 records within the same survey year, MEPS 2017–2023. Open symbols show descriptive annual estimates. Confidence intervals are omitted because sparse annual samples resulted in unstable interval estimation. Connecting lines are visual guides and do not establish temporal trends. Annual denominators ranged from 6 to 30 person‐years, and all annual medication‐user counts were fewer than 30. Independent E11/I63 definition: 125 person‐years. All annual medication counts are fewer than 30. Descriptive estimates only; confidence intervals omitted because of sparse, unstable estimates. Annual denominators (2017–2023): 30, 22, 17, 12, 22, 16, 6. Estimates are highly imprecise. *GLP‐1RA group includes dual GIP/GLP‐1 receptor agonists. Panel scales differ.

**FIGURE 6:**
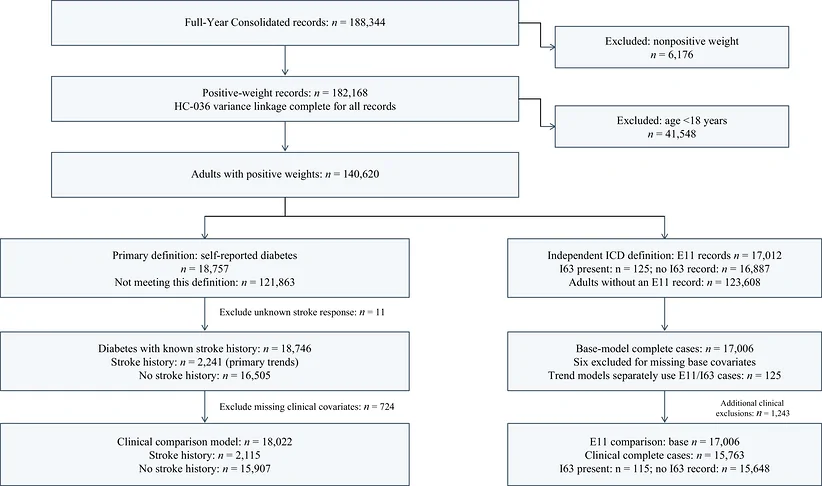
Adjusted associations between annual I63 records and glucose‐lowering medication use among adults with E11 records, MEPS 2017–2023. Points represent adjusted odds ratios, and horizontal lines represent 95% confidence intervals. The reference group comprises adults with E11 records without an I63 record during the same survey year. Base models adjusted for age, sex, race and ethnicity, insurance status, poverty category, and survey year; expanded clinical models additionally adjusted for coronary disease history, annual recorded chronic kidney disease, heart failure, obesity, and approximate diabetes duration. Absence of an annual I63 record does not establish absence of a history of stroke. Exploratory alternative‐definition analysis. Absence of an I63 record does not establish absence of stroke history. Only 125 exposed person‐years (115 in the clinical model); interpret sparse outcomes cautiously. *Includes dual GIP/GLP‐1 receptor agonists. Thiazolidinedione use was recorded in only three exposed person‐years.

In the 2018–2023 primary‐cohort analysis, increases in the GLP‐1 receptor agonist group, SGLT2 inhibitors, and their composite remained evident. Any‐medication and metformin trends were statistically significant in this shorter window, unlike in the full‐period analysis, demonstrating sensitivity of some findings to the chosen time window (Table 14).

**TABLE 14:** Additional trend analysis restricted to 2018–2023.

Medication	OR/year (95% CI)	*p*	BH q
Any glucose‐lowering medication	0.89 (0.81–0.97)	0.011	0.025
Metformin	0.92 (0.86–0.99)	0.023	0.042
Insulin	0.96 (0.89–1.04)	0.320	0.360
GLP‐1RA group^*^	1.26 (1.12–1.42)	< 0.001	< 0.001
SGLT2 inhibitors	1.52 (1.32–1.75)	< 0.001	< 0.001
DPP‐4 inhibitors	0.88 (0.77–1.00)	0.053	0.080
Sulfonylureas	0.95 (0.86–1.04)	0.254	0.326
Thiazolidinediones	1.04 (0.80–1.34)	0.777	0.777
GLP‐1RA group^*^ or SGLT2 inhibitor	1.40 (1.26–1.55)	< 0.001	< 0.001

*Note: N* = 1900 primary‐cohort person‐years. This post hoc time‐window analysis excludes 2017, preceding changes in condition collection; it is not an alternative‐case‐definition analysis. Any‐medication and metformin trends differ in significance from the full‐period results, so robustness is not claimed for every outcome.

## 4 Discussion

In this nationally representative analysis, approximately four in five US adults with diabetes and stroke used a glucose‐lowering medication during 2017–2023. Metformin remained the most common agent, over one‐third used insulin, nearly one‐quarter used sulfonylureas, and about one in six person‐years had records of three or more distinct medication classes within the year. The most notable temporal shift was the rapid rise in GLP‐1RA and SGLT2i use; the estimated downward trend in DPP‐4i use did not reach statistical significance. By 2023, approximately one‐third of this population had a recorded GLP‐1RA or SGLT2i prescription. This estimate describes medication use but does not establish the proportion eligible for these therapies or whether nonuse represented an unmet treatment need.

Biological and functional considerations provide clinical context for these medication‐use patterns. Hyperglycemia and insulin resistance contribute to vascular injury through endothelial dysfunction, oxidative stress, inflammation, and prothrombotic pathways (Lodi et al. 2026). Stroke‐related impairments may complicate diabetes management (Yang et al. 2021). These mechanisms were not evaluated in the present analysis and should not be interpreted as explanations established by the observed medication‐use patterns.

The rise in GLP‐1RA use has particular relevance for cerebrovascular prevention. Randomized trial evidence provides relevant context, although the cerebrovascular findings differ across drug classes. An exploratory analysis of REWIND reported a lower risk of stroke with dulaglutide (Gerstein et al. 2020). A meta‐analysis of SGLT2 inhibitor trials did not demonstrate a significant reduction in total stroke (Pasqualotto et al. 2024). These findings do not establish equivalent stroke‐prevention effects across classes or explain medication selection in the present cohort. Mechanisms extend beyond glycemic control to include weight reduction, blood pressure lowering, improved endothelial function, and reduced inflammation (Gala et al. 2025). The increase in GLP‐1RA use occurred during a period of evolving cardiovascular evidence and guideline recommendations. However, this analysis did not evaluate whether guideline changes, coverage restrictions, costs, tolerability, or functional limitations contributed to the observed trends. SGLT2i use also increased substantially, but the reasons for this increase could not be determined from the present analysis. Yet pooled trial data show neutral associations with total and ischemic stroke despite major cardiorenal benefits (Tsai et al. 2023). Thus, while combining GLP‐1RAs and SGLT2is serves as a useful marker of contemporary therapy uptake, it does not imply equivalent stroke protection across agents. Future studies incorporating treatment indications and detailed clinical characteristics could help distinguish changes in medication use from changes in treatment eligibility.

The lower pooled prevalence of GLP‐1RA and SGLT2i use compared with insulin should be interpreted in the context of potentially different treatment indications and patient characteristics. Diabetes severity, comorbidities, contraindications, tolerability, affordability, and patient preferences may influence medication selection. However, these factors were not comprehensively assessed, and the present analysis cannot determine their contribution to the observed medication mix.

Although the estimated trend in DPP‐4i use was downward during the period of increasing GLP‐1RA and SGLT2i use, it was not statistically significant. Medication selection should consider glycemic efficacy, cardiovascular and kidney disease, hypoglycemia risk, weight effects, treatment burden, and access, as outlined in treatment guidelines (ElSayed et al. 2023). These population‐level trends cannot establish whether individual patients switched from DPP‐4is to GLP‐1RAs or SGLT2is. The associations of stroke history with metformin and insulin use were attenuated after additional clinical adjustment. The metformin association was no longer statistically significant, whereas the insulin association remained nominally significant but did not meet the exploratory multiplicity threshold. These findings indicate that the comparisons depend partly on the covariates included in the model, but they do not establish why individual medications were selected. The estimates should therefore be interpreted as adjusted associations rather than effects attributable to stroke history, and they cannot establish overtreatment or undertreatment. Hypoglycemia risk remains relevant to individualized treatment selection, particularly for regimens containing insulin or sulfonylureas (Demirbilek et al. 2023). However, this study did not measure hypoglycemic events or determine the safety of individual treatment regimens. When reviewing medications in adults with diabetes and a history of stroke, clinicians may consider whether glycemic targets are appropriate, regimens can be simplified, or lower‐risk alternatives can replace insulin secretagogues without compromising affordability.

Thiazolidinedione use was uncommon. The IRIS trial demonstrated a reduction in stroke or myocardial infarction with pioglitazone among patients without diabetes who had insulin resistance and a recent ischemic stroke or transient ischemic attack (Kernan et al. 2016). Because this population differed from our cohort, low thiazolidinedione use alone cannot establish underuse of an indicated treatment. Adjusted analyses did not show greater GLP‐1RA/SGLT2i use in stroke survivors than in diabetic adults without stroke. This comparison does not establish a quality‐of‐care gap because treatment eligibility and the reasons for medication selection were not determined. The relevant question is whether each patient receives an informed, phenotype‐directed regimen after evaluating vascular risk, organ status, hypoglycemia risk, and self‐management capacity. The alternative E11/I63 definition identified a small population that overlapped only partially with the primary cohort. Repeated trend estimates were imprecise, and some adjusted associations differed from the primary results. Differences in case ascertainment and comparison‐group definitions, together with sparse outcome counts, limit direct comparisons between the analyses. The alternative definition should therefore not be interpreted as a more accurate subset or as validation of the primary findings.

These findings carry implications for practice and research. Stroke care pathways should include explicit diabetes medication reconciliation. Neurologists, primary care clinicians, endocrinologists, and rehabilitation teams should share responsibility for individualized regimens. Future research should evaluate medication access and clinical appropriateness directly rather than infer them from class‐specific use alone. Future studies should link prescribing data to clinical outcomes, including recurrent stroke, hypoglycemia, and functional recovery. This study has several strengths, including seven years of nationally representative data, comprehensive drug class coverage, and appropriate survey weighting. Limitations include the cross‐sectional design, self‐reported diagnoses, absence of HbA1c and measured kidney function, reliance on approximate diabetes duration, exclusion of institutionalized adults, and sparse medication‐class counts in some analyses. Annual N18, I50, and E66 records captured conditions recorded during the survey year rather than clinically validated comorbidities. Absence of a corresponding code did not establish absence of disease, and E66 records did not provide a BMI‐based measure of obesity. Residual confounding may therefore remain despite clinical adjustment. The primary cohort was based on self‐reported diabetes and a history of stroke, without reliable information on diabetes type or stroke subtype, timing, severity, or recurrence. We therefore could not determine the temporal relationship between stroke and medication use or evaluate treatment initiation or modification following a stroke. The findings should be interpreted as annual medication‐use patterns in this broadly defined population rather than as an assessment of subtype‐specific or post‐stroke treatment strategies. Annual medication‐class counts could not distinguish concurrent combination therapy from sequential switching because treatment overlap was not established in this analysis. Multiple person‐years may arise from overlapping survey panels, so estimates reflect average annual prevalence rather than incident cohorts. Despite these limitations, this analysis provides a contemporary population‐level view of treatment patterns in a high‐risk group during a period of rapid therapeutic evolution. The alternative ICD‐based cohort was small, with sparse medication‐class counts that limited the precision and stability of the trend and adjusted comparison analyses. Small annual survey domains also resulted in unstable confidence interval estimation; annual percentages were therefore reported descriptively and should not be used to infer year‐to‐year changes. No additional subgroup analyses were conducted in this cohort.

## 5 Conclusion

From 2017 to 2023, GLP‐1RA and SGLT2i use rose substantially among US adults with diabetes and a history of stroke. Metformin remained the most commonly used agent, while evidence of higher insulin use among adults with a history of stroke was not conclusive after clinical adjustment and exploratory correction for multiple comparisons. These findings suggest growing adoption of newer therapies and describe the annual distribution of recorded medication classes. Medication review in adults with diabetes and a history of stroke should consider hypoglycemia risk, treatment burden, and access. Whether these changes in medication use are associated with clinical outcomes requires further investigation.

## Author Contributions

Y.J.C. conceived and designed the study, acquired the data and drafted the manuscript. T.L. analyzed the data. Y.J.X. contributed to the interpretation of the results and critical revision of the manuscript for important intellectual content. Y.J.C. developed the software and provided technical support. T.L. had the primary responsibility for final content. All authors have read and approved the final manuscript.

## Funding

This study was supported by Zhejiang Provincial Health Industry Science and Technology Program (2025HY1336).

## Ethics Statement

This study drew on de‐identified information from the Medical Expenditure Panel Survey, conducted under the auspices of the Agency for Healthcare Research and Quality. No individually identifiable information was utilized.

## Conflicts of Interest

The authors declare no conflicts of interest.

## Data Availability

The data used in this study are publicly available from the Agency for Healthcare Research and Quality Medical Expenditure Panel Survey (MEPS) data download portal (https://meps.ahrq.gov/mepsweb/data_stats/download_data_files.jsp). We used the 2017–2023 Household Component Full‐Year Consolidated, Prescribed Medicines, and Medical Conditions files, together with the HC‐036 1996–2023 Pooled Linkage Variance Estimation File (h36u23). The annual files can be located by survey year and file type, and the pooled linkage file by public use file number HC‐036.
